# Global thrombosis test for assessing thrombotic status and efficacy of antithrombotic diet and other conditions

**DOI:** 10.2144/fsoa-2021-0086

**Published:** 2022-01-31

**Authors:** Masahiro Murakami, Kazunori Otsui, Yoshinobu Ijiri, Muneshige Shimizu, Hideo Ikarugi, Wataru Shioyama, Junichiro Yamamoto, Kjell S Sakariassen

**Affiliations:** 1 Faculty of Pharmacy, Osaka Ohtani University, Osaka, 584 8540, Japan; 2 Department of General Internal Medicine, Kobe University Hospital, Kobe, 650 0017, Japan; 3 Department of Health & Nutrition, Osaka Shoin Women’s University, Osaka, 577 8550, Japan; 4 Department of Fisheries, School of Marine Science & Technology, Tokai University, Shizuoka, 424 8610, Japan; 5 School of Economics & Management, University of Hyogo, Kobe, 651 2197, Japan; 6 Department of Internal Medicine, Division of Cardiovascular Medicine, Shiga University of Medical Science, Shiga, 520 2192, Japan; 7 Kobe Gakuin University, Kobe, 651 2180, Japan; 8 Biella, BI, I 13900, Italy

**Keywords:** antithrombotic vegetables, cancer associated thrombosis, cardiovascular disease, COVID-19, exercise, fibrinolysis, native blood, platelet aggregation, shear stress, stroke

## Abstract

Because of the high mortality from myocardial infarction and stroke, there is a great demand for finding novel methods of diagnosis, prevention and treatment of these diseases. Most of the current tests measure important determinants of thrombosis such as platelet function, coagulation and fibrinolysis in isolation; therefore, a global test measuring the actual thrombotic status would be more useful in clinical conditions. We obtained considerable experience by using the global thrombosis test, which determines the actual thrombotic status by taking into account the measured platelet reactivity, coagulation and fibrinolytic activities. In animal experiments, we found significant correlation between the *ex vivo* global thrombosis test measurements and the *in vivo* thrombotic status. The published evidence for the benefit of an antithrombotic diet with regular physical exercise is also described.

## Plain language summary

There is a great concern in the general population how to detect the risk of thrombotic events and prevent the high mortality from stroke, myocardial infarction, sudden death and cancer-associated thrombosis. Our experience on antithrombotic fruits and vegetables intake and regular exercise assessed by the global thrombosis test suggested a potentially unique way of preventing these life-threatening diseases. In addition, global thrombosis testing may offer some benefit in detecting risk of thrombotic of forthcoming thrombotic events in cancer and COVID-19 virus-infected patients.

## The use of an overall *in vivo* thrombosis/thrombolysis test in experimental animals

Because of the severity of thromboembolic diseases such as myocardial infarction and stroke, there is a great demand for finding way of prevention and treatment of such diseases. For this purpose, finding a pathologically relevant tests suitable for the assessment of the actual thrombotic status is crucially important. At present, various tests exist claiming to assess determinants of thrombosis such as platelet reactivity, coagulation and fibrinolysis in isolation, but the common shortcoming of these test is the lack of relevance to the pathology of arterial thrombotic diseases. Most of these tests measure platelet function to various thrombotic stimuli and as such suitable for monitoring antiplatelet therapy while these tests do not detect endogenous fibrinolysis. In the latter field, d-dimer measurement is the predominant laboratory test [1–8]. Finding a test which assesses reactivity of platelets, coagulation and fibrinolysis is justified by the ‘Virchow’s triad’ demonstrating the complexity of the interaction between platelets, other blood cells, vessel wall and blood flow in thrombus formation and lysis. This recognition requires the use of a global test, which takes into account of all contributors to the thrombotic process. For these reasons we employed the global thrombosis test (GTT) in animal experiments, where the conditions are reproducible and can be standardized.

Numerous animal models of thrombosis and thrombolysis (fibrinolysis) exist. Yamamoto and colleagues have employed helium–neon (He–Ne) laser-induced thrombosis/fibrinolysis model in rats and mice since the year 1989 [9–20]. Ruby biolaser-induced thrombosis model was first published by Arfors and colleagues [21], followed by He–Ne laser-induced thrombosis model in rodents by Kovacs, Gorog and colleagues [22–26]. In our experience the He–Ne laser-induced animal thrombosis model is the most reliable technique providing reproducible and reliable results.

**Figure 1.**
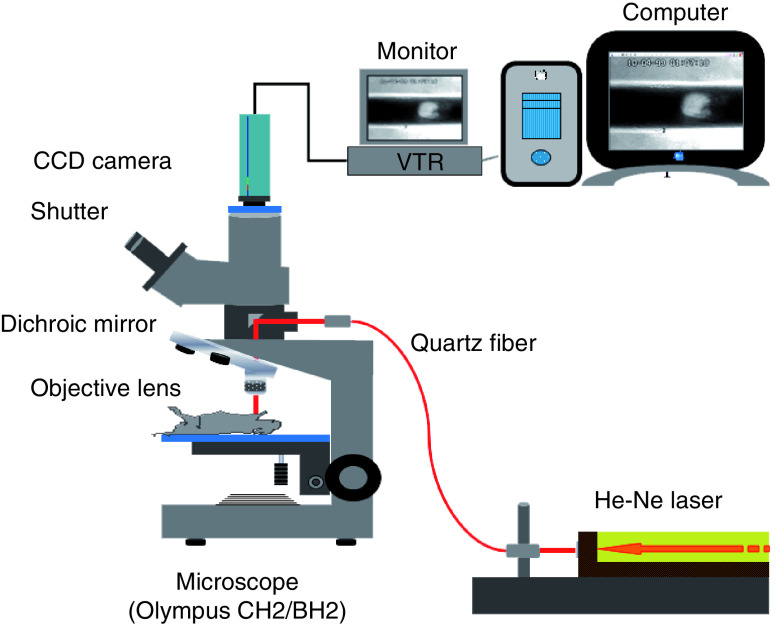
Helium–neon laser-induced thrombosis/thrombolysis (fibrinolysis) system. Figure taken from Figure 1 from [35] (original article is open access [CC-BY]). CCD: Charged-coupled device.

**Figure 2.**
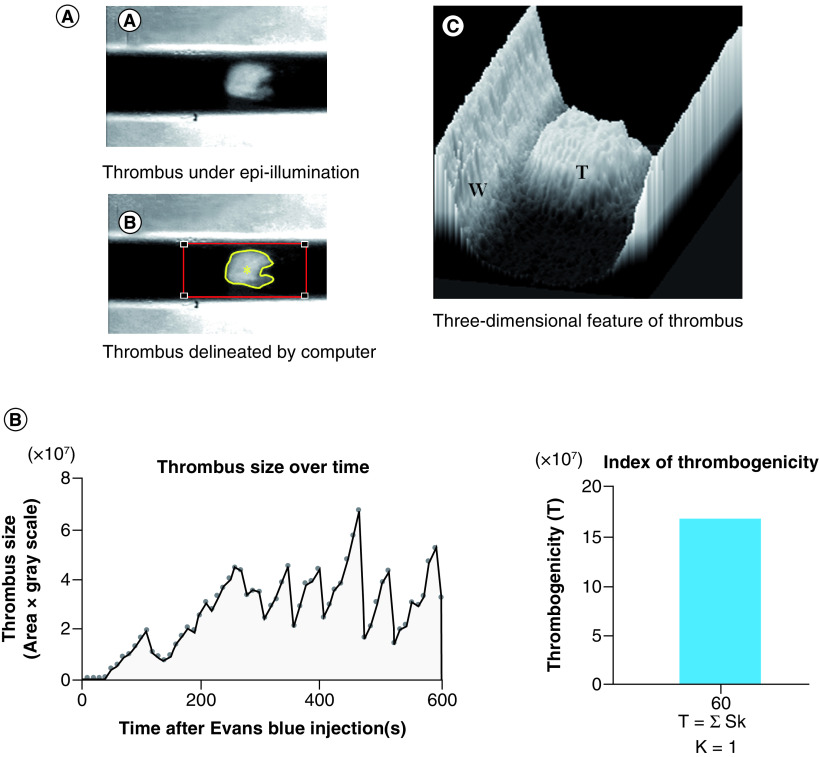
Thrombosis in mouse carotid artery. **(A)** Thrombus size measurement of thrombus in mouse carotid artery. **(B)** Index of thrombogenicity measurement. Figure taken from Figure 1 from [35] (original article is open access [CC-BY]). T: Thrombus; W: Vessel wall.

**Figure 3.**
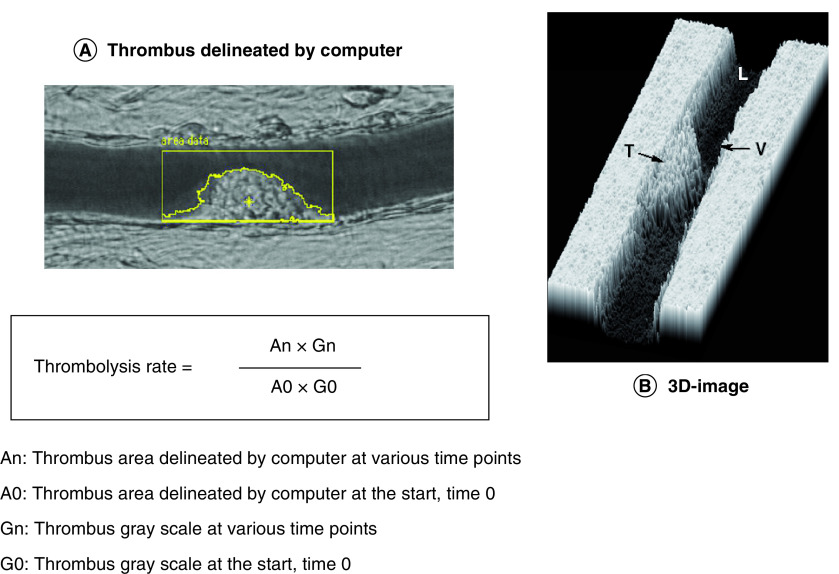
Thrombolysis measurement in rat mesenteric microvessel. Thrombus area is calculated by delineating thrombus using computer **(A)**. Subsequently, thrombus size **(B)** is obtained by multiplying gray scale and the area. Thrombolysis rate is compared with that at the start. L: Lumen; T: Thrombus; V: Vessel wall. Figure reproduced with permission from [32] (Copyright © 2001, © 2001 S. Karger AG, Basel).

Earlier Furie and coworkers used the He–Ne laser-induced thrombosis model in mice to analyze thrombotic mechanism at molecular level [27–29]. He–Ne laser-induced thrombosis/fibrinolysis animal model is shown in Figure 1. In brief, the mesenteric or pial microvessel of anesthetized rat or the carotid artery of anesthetized mouse was exposed, and Evans blue dye was injected through the veins and then the exposed blood vessel was irradiated with laser beam. The injected dye specifically absorbs the laser energy, converts it to heat and as such burns the blood vessel from inside inducing thrombus formation. Thrombus formation in rat mesenteric vessels or in the carotid artery of mice having irradiated by laser was video monitored (https://drive.google.com/file/d/1EMaD-Rwt_lDlc_99rwy_q2WeSIWaX-wF/view?usp=sharing); (https://drive.google.com/file/d/1VNMjAjbQFRF18ZgdxEsRSzqpFX0gyh-W/view?usp=sharing). Severity of thrombotic reaction in the irradiated rat mesenteric microvessel was expressed by the number of irradiations required to cause complete occlusion of blood flow. In mouse carotid artery, the video recording in every 10 s in the first 10 min after irradiation was performed and the total size of emboli following irradiation was measured (Figure 2). In thrombolytic activity measurement, the size of mural thrombus formed at the irradiation site was calculated with the formula shown in Figure 3 [30–34].

## Overall *ex vivo* thrombosis/thrombolysis (fibrinolysis) test in experimental animals & in humans

Tests to assess thrombotic status using native (nonanticoagulated) blood and high shear forces were established by Baumgartner *et al.* and Sakariassen *et al.* [35–41]. Their original shear rate dependent thrombosis chambers are most useful for investigating human thrombotic mechanisms under blood flow conditions and for monitoring human antithrombotic drugs. The haemostatometer were invented by Kovacs and Gorog to measure high shear-induced thrombosis and the subsequent thrombolysis [42–46]. The haemostatometer and GTT tests, in which platelets were activated solely by high shear, proved to be useful assessing thrombus formation under high shear condition and proved to be clinically useful in assessing thrombotic status of patients. These tests were also suitable for selecting fruits and vegetables having antithrombotic activity for use as an antithrombotic diet in humans [47–53].

## Description of GTT test

**Figure 4.**
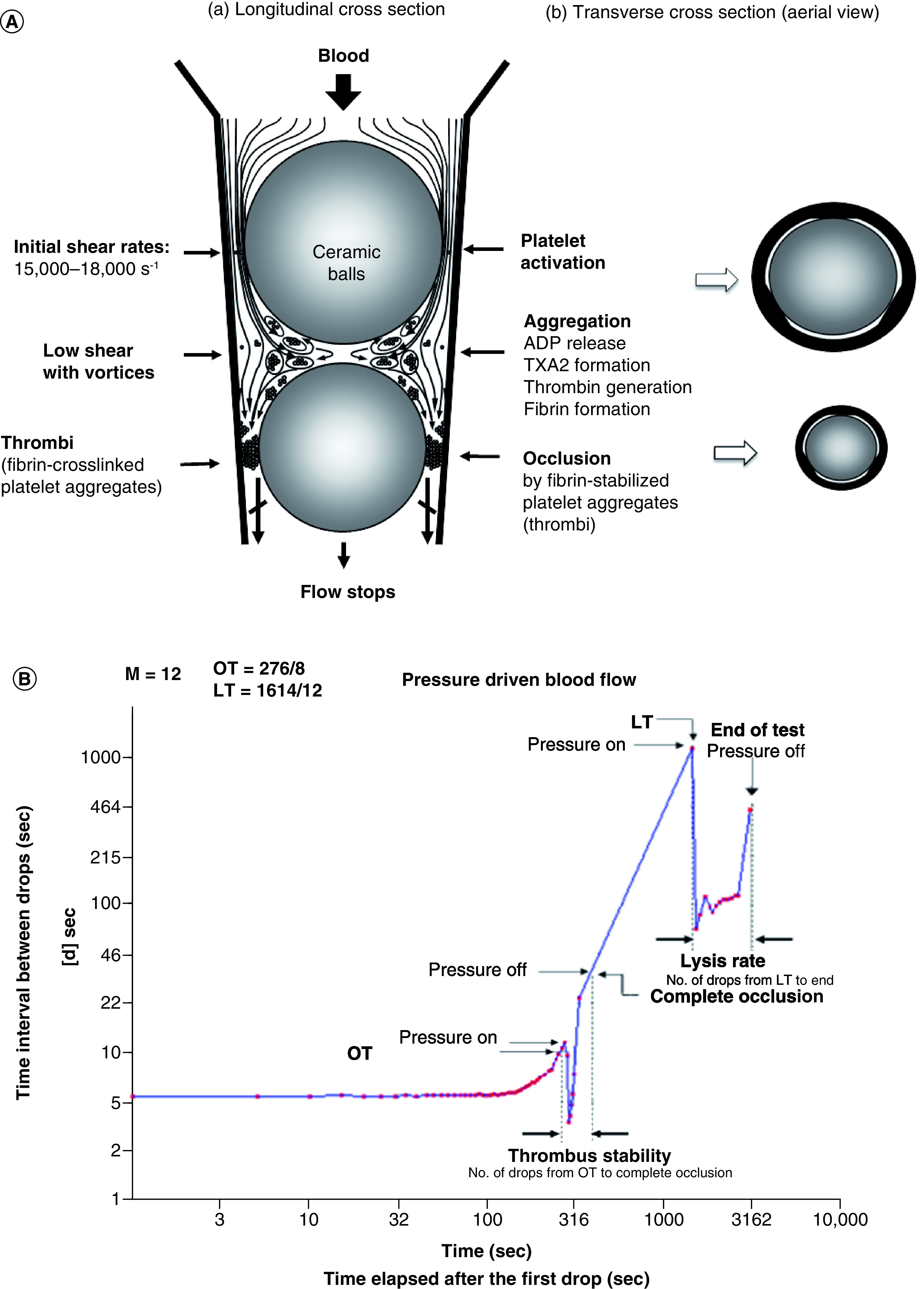
Global thrombosis test. **(A)** Principle of *ex vivo* GTT. Platelets are activated under high shear condition at the upper gaps created along the inner of a conical plastic tube. Activated platelets form fibrin-stabilized platelet aggregates under low shear condition between two balls. Fibrin-stabilized platelet aggregates occlude the lower gaps. **(B)** Pattern obtained by GTT (GTT-3). GTT: Global thrombosis test; LT: Lysis time OT: Occlusion time.

GTT test tube has a conical section in which two ceramic ball bearings are placed (Figure 4A). Because of three flat segments formed on the inner surface of the tube, three narrow gaps exist adjacent to the ball bearings. When blood is added to the tube, it flows through the narrow gaps by the ball bearings and the droplets are collected in a reservoir downstream. While passing through the gaps by the upper (larger) ball bearing, platelets are exposed to high shear stress and become activated. In the space between the two ball bearings, platelet aggregates and thrombin is generated from the activated platelets. As fibrin-stabilized thrombi reach the lower ball bearing, blood flow gradually reduced and finally arrested. The instrument detects the time interval (d, s) between two consecutive blood drops falling into the reservoir. At the start of the test, flow is rapid and hence (d) is small. Subsequently, the flow rate decreases and hence (d) increases. When the actual default d ≥15 s is reached, this time is displayed as occlusion time (OT s). Subsequently the flow is completely arrested. After OT, a preset ‘thrombi stabilization period’ follows during which the sensors ignore counting the blood drops. This time allows stabilization of the fully occlusive thrombi. Eventually, due to endogenous thrombolysis (fibrinolysis), flow is partially restored as indicated by detection of the first blood drop after OT. This is recorded as lysis time (LT s). Typical pattern is shown in Figure 4B.

GTT3 test starts by placing disposable test tube into the heated channel of the instrument and setting the instrument in standby mode, waiting for the blood sample to start the measurement (URL: https://drive.google.com/file/d/1Up-MgDCFPEBWYN3hKrmCobMCm47XpTp8/view). Blood is withdrawn from the antecubital vein, while the tourniquet constriction should be limited to a short time, only while the needle is inserted into the vein. To avoid activation of platelets and coagulation during withdrawal of blood, the ‘two-syringe blood taking technique’ is used. The first withdrawn 3–4 ml blood is used for laboratory tests and only the 4 ml blood in the second syringe is used for GTT measurement. The nonanticoagulated blood sample must be transferred from the syringe into the GTT test tube within <15 s from the withdrawal. With the transfer of blood into the test tube, test starts automatically and along the measurement reaching OT/thrombus stability then LT/rate of thrombolysis, these data are displayed and simultaneously recorded in an SD card for the possibility of displaying the measurement in a graph form later by a special algorithm ‘GTT-Draw’ [54–62].

## Thrombotic status is determined by the balance between thrombotic & fibrinolytic activities

**Figure 5.**
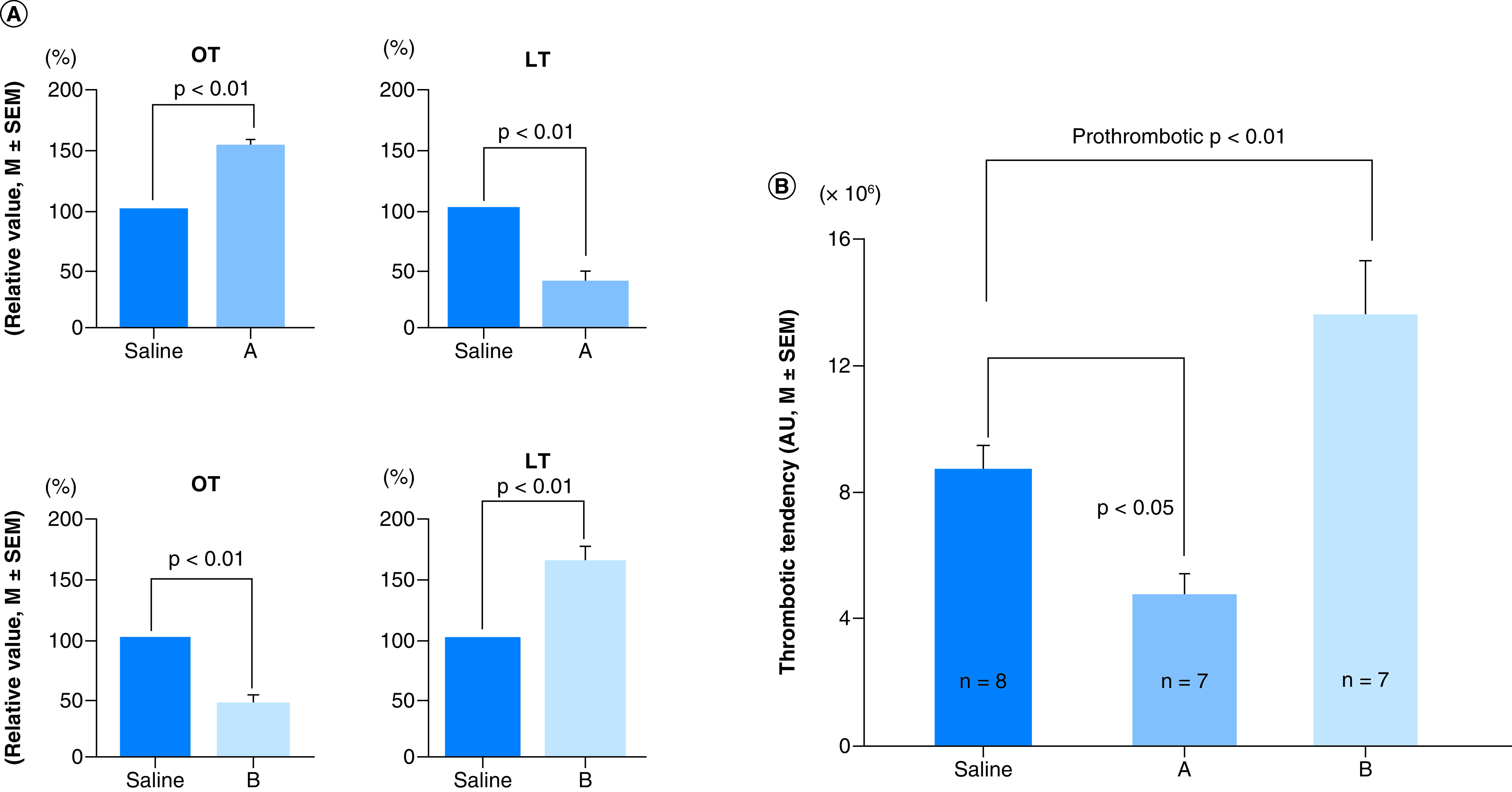
*Ex vivo* and *in vivo* test. **(A)** Assessment of antithrombotic/prothrombotic activity by *ex vivo* test (GTT). **(B)** Assessment by *in vivo* test (He–Ne laser-induced thrombosis test). **(A)** Cabernet sauvignon. **(B)** Neo muscat. (Figure taken from Figure 1 from [50] (original article is open access [CC-BY]). GTT: Global thrombosis test; LT: Lysis time OT: Occlusion time.

The importance of balance between thrombotic and fibrinolytic activities determining the overall thrombotic status was confirmed in our experiments testing various fruit and vegetable varieties for antithrombotic activity. First, thrombotic (OT) and fibrinolytic activities (LT) of fruit and vegetables were measured by *ex vivo* GTT using rat blood. Next, the predicted antithrombotic activity was confirmed by *in vivo* He–Ne laser-induced thrombosis test in mice. These results are shown in Figure 5. Grape varieties were classified as antithrombotic and prothrombotic varieties. Cabernet sauvignon (A) increased OT suggesting antithrombotic activity and decreased LT suggesting antithrombotic activity. The overall results showed being antithrombotic. On the contrary, Neo muscat (B) decreased OT suggesting prothrombotic activity and increased LT suggesting prothrombotic activity (Figure 5A). These predictions were demonstrated by He–Ne laser-induced thrombosis test (Figure 5B [50]). The same *ex vivo* and *in vivo* correlation has been described in carrot varieties [49]. Similar correlation was shown in congenital diabetic prothrombotic Goto-Kakizaki rat and antithrombotic Otsuka Long Evans Tokushima Fatty rat [63,64].

## Contribution from endothelium to thrombosis

Prothrombotic status of spontaneously hypertensive stroke prone rats was confirmed by the *in vivo* laser-induced thrombosis test. In contrast, platelet reactivity measured by *ex vivo* shear-induced thrombosis test using native blood was inhibited rather than enhanced [65,66]. To look into this contradiction, contribution from endothelium to thrombosis was examined *in vivo* in mice using the flow-mediated vasodilation test. Western-style high-fat diet and Japanese-style low fat diet were given to mice and thrombotic status was measured by the *in vivo* laser-induced thrombosis test. Results show that high-fat diet caused prothrombotic condition. High-fat diet did not affect the *ex vivo* shear-induced thrombosis test but induced endothelial dysfunction [67–71]. Based on our finding, we suggested to couple the GTT test with the endothelial function evaluation using the flow-mediated vasodilation test.

## Comparing thrombotic status of healthy people & cancer patients in different countries & under different conditions

**Figure 6.**
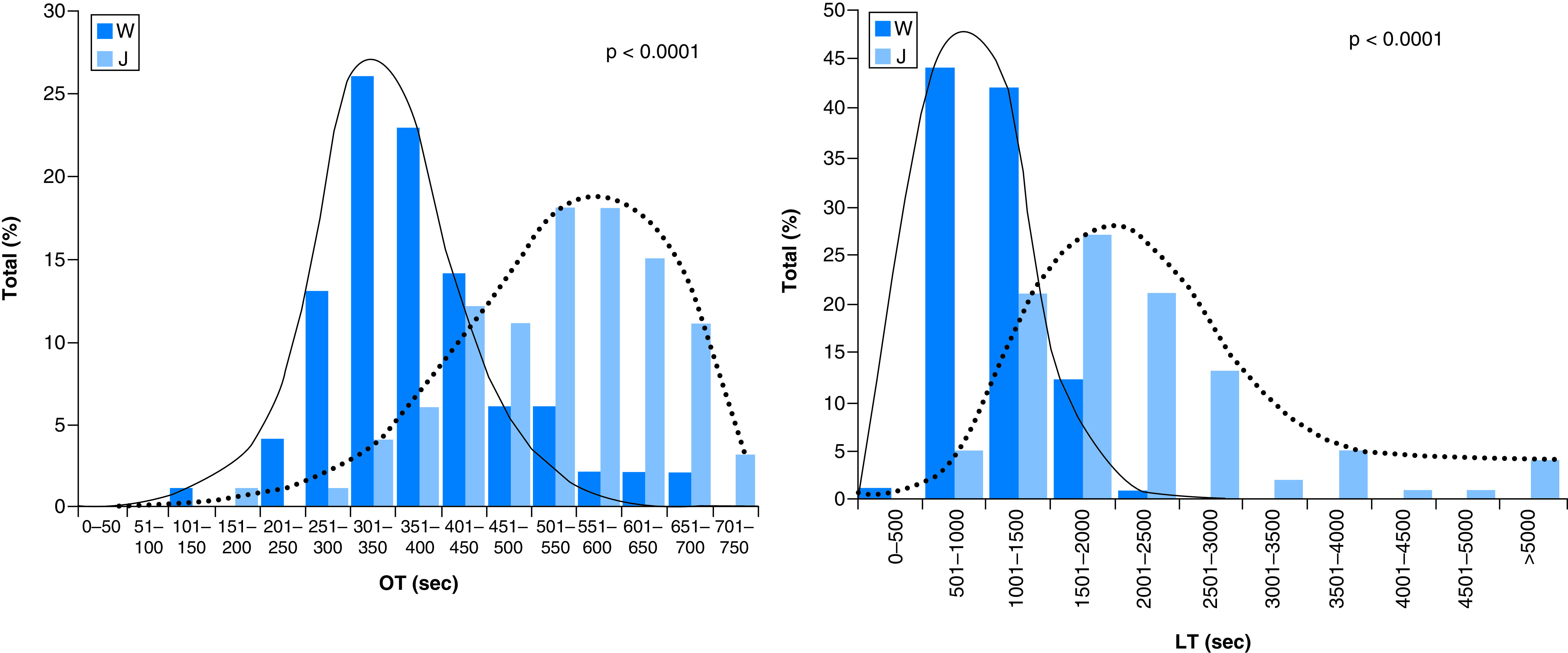
Comparison of thrombotic status between different races. Thrombotic and fibrinolytic activities in Westerners and Japanese, measured by GTT. GTT: Global thrombosis test; J: Japanese; LT: Lysis time OT: Occlusion time; W: Westerners. Reprinted from [72] with permission from Elsevier.

OT as measured by GTT in British people is shorter than that of Japanese and LT of British is shorter than that of Japanese. This finding shows that in British subject thrombus forms rapidly but dissolved quickly, while in Japanese people thrombus forms and dissolved at a lower rate [72]. People’s thrombotic status appears to be different in different countries, and for this reason epidemiological survey is necessary to have respective standard OT and LT (Figure 6). Aging and habitual smoking enhance thrombotic status through inhibiting spontaneous fibrinolytic activity. This was obvious in the elderly [73,74]. Overwork of doctors at overnight causes prothrombotic status due to enhancing thrombosis and inhibiting fibrinolysis. This was not detected by APTT, PT-INR and PAI-1 measurements [75].

**Figure 7.**
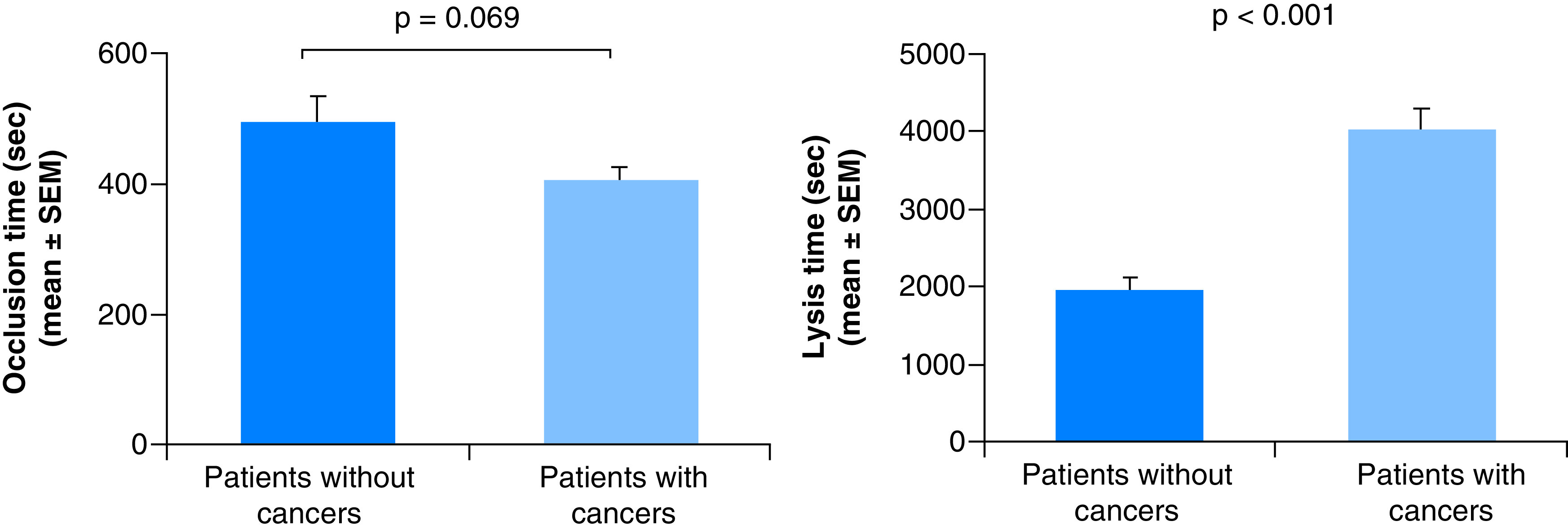
Thrombotic status of patients with and without cancers. Enhanced thrombotic status in patients with cancers. Figure taken from [76]; was orally presented by Dr. Shioyama at *The 25th Kinki Thrombosis Research Society Meeting. Osaka, Japan (2020)*.

The cause and mechanism of cancer-associated thrombosis has been investigated over the years. Thrombotic status of patients with cancers and that without cancers are shown in Figure 7. OT of patients with cancers (n = 48) was slightly shorter than that of patients without cancer (n = 17) but the difference did not reach significant level (p = 0.069). This might be due to the difference in number between the two groups. On the contrary, LT in patients with cancers were significantly prolonged (p < 0.0001). Overall thrombotic status of patients with cancers is higher, they are more prothrombotic than that of patients without cancers [76]. GTT measurement may be helpful in finding the most effective treatment of thrombotic complications in cancer patients. As distribution patterns of OT and LT are different in people from different countries, GTT testing and monitoring therapy would offer personalized medication.

## Prevention of thrombotic diseases by daily intake of antithrombotic fruits, vegetable varieties & physical exercise

Antithrombotic effect of Mediterranean diet and various fruits and vegetables has been reported. Results of investigation aimed to find the specified ingredients responsible for the antithrombotic effect were also published [77–86]. We were the first reporting that the antithrombotic effect of fruits and vegetables is dependent on the varieties, but not on species [49–52]. GTT enables to classify fruit and vegetable varieties to antithrombotic and prothrombotic ones. Short- or long-term intake of antithrombotic fruits and vegetable varieties decreases thrombotic status in humans [87,88]. Further, we confirmed that the antithrombotic effect of fruits and vegetables are independent from antioxidants and polyphenols [49–51].

Government authorities generally recommend physical exercise to prevent stroke and cardiovascular disease, but sometimes exercise causes sudden death (exercise paradox) [89–99]. Yamamoto *et al.* suggested that regular measurement of thrombotic status of individuals and doing exercise in intensity and time length well-matched to individuals are beneficial. Acute strenuous exercise significantly shortens OT, but long term regular mild exercise increases OT. There was no significant difference in the rate of fibrinolysis between the two types of exercise [100–102]. These results suggest that acute and strenuous exercise may be dangerous and may cause sudden death. In contrast, long term regular mild exercise may be beneficial by having antithrombotic effect.

## Supplement

### Coronavirus associated thrombosis

Thromboprophylaxis in coronavirus (COVID-19) pandemic seems to be justified [103]. In addition, vaccine-induced immune thrombocytopenia was also reported [104] which suggests a prothrombotic condition. The cause and mechanism of thromboembolic episodes associated with COVID-19 and vaccination are not clear, blood cells (erythrocytes, leucocytes, platelets) and endothelial cells may be involved. Currently the most recommended laboratory tests to detect prothrombotic condition are the d-dimer, PT, APTT, fibrinogen and platelet count measurements [105–107]. Non-plasmin enzymes may also be involved in fibrinolysis *in vivo* [108] Limitations of testing these biomarkers suggest the use of global thrombotic status measurement in people infected with COVID-19. The clinical benefit of current tests like thromboelastography and d-dimer measurements still needs to be established [109,110]. In testing nonanticoagulated whole blood with the GTT test in normal and cardiac patients, Yamamoto, Kovacs, Gorog and colleagues have demonstrated that leucocytes play a significant role in thrombosis/fibrinolysis [111]. GTT using nonanticoagulated whole blood is suitable to assess thrombotic status and guide antithrombotic medication in COVID-19-associated pulmonary thromboembolic disorders.

## Limitations of tests using native blood

Starting the GTT test within 15–20 s after withdrawal of blood samples requires the presence of highly trained, experienced phlebotomist, the GTT instrument and the patient at the site of test. Such conditions are difficult to fulfill in the present system of laboratory testing in hospitals. Video shown in this article may be helpful for those who want to use GTT in clinical practice.

## Conclusion & future perspective

Adopting a long-term use of an experimentally proven antithrombotic diet and monitored regular physical exercise to put a stop to arterial thrombotic disorders, is certainly an interesting and challenging possibility. For this approach to be successful, it is important to find a test or tests that can monitor thrombotic status under pathologically relevant conditions. Compared with the common platelet function or coagulation tests currently being used, the shear-induced global thrombosis and fibrinolysis test (GTT) performed with native (nonanticoagulated) blood is suitable for the concept. Now that the pathologically relevant tools of testing and monitoring thrombotic status of individuals have been found, further investigations need to focus on the organization of large-scale and monitored trials that include both healthy individuals and those at risk of atherothrombotic diseases and cardiovascular events. The outcome of such trials may justify the everyday consumption of an antithrombotic diet with regular physical exercise, and this would be a simple and economical way of prevention of arterial thrombotic diseases.

**Executive summary**

- To date, global thrombosis test (GTT) is the most suitable test for assessing thrombotic status of patients with thrombotic disorders.
- Interest in prevention of thrombotic disorders by antithrombotic vegetable varieties and physical exercise is increasing. A pathologically relevant point-of-care global tests for the assessment of global thrombotic status is needed.
- The GTT enables the simultaneous measurement of thrombosis and endogenous fibrinolysis under high shear stress. This technique proved to be suitable for screening fruits and vegetables for antithrombotic effect, making possible to establish an antithrombotic diet and also to individualize the need of physical exercise in people at risk of thrombotic events.
- GTT-monitored large-scale trials are needed to verify the beneficial effect of an antithrombotic diet with regular physical exercise in the prevention of arterial thrombotic events.
- The GTT may clarify the mechanism of association of thrombotic events and cancer and help in the treatment of COVID-19-associated thromboembolic disorders.
